# Surface Assembly of DNA Origami on a Lipid Bilayer Observed Using High-Speed Atomic Force Microscopy

**DOI:** 10.3390/molecules27134224

**Published:** 2022-06-30

**Authors:** Masayuki Endo

**Affiliations:** 1 Organization for Research and Development of Innovative Science and Technology, Kansai University, Suita, Osaka 564-8680, Japan; endo@kansai-u.ac.jp; Tel.: +81-6-6368-1111; 2 Institute for Integrated Cell-Material Sciences, Kyoto University, Sakyo, Kyoto 606-8501, Japan

**Keywords:** DNA origami, DNA nanotechnology, surface assembly, lipid bilayer, high-speed AFM

## Abstract

The micrometer-scale assembly of various DNA nanostructures is one of the major challenges for further progress in DNA nanotechnology. Programmed patterns of 1D and 2D DNA origami assembly using specific DNA strands and micrometer-sized lattice assembly using cross-shaped DNA origami were performed on a lipid bilayer surface. During the diffusion of DNA origami on the membrane surface, the formation of lattices and their rearrangement in real-time were observed using high-speed atomic force microscopy (HS-AFM). The formed lattices were used to further assemble DNA origami tiles into their cavities. Various patterns of lattice–tile complexes were created by changing the interactions between the lattice and tiles. For the control of the nanostructure formation, the photo-controlled assembly and disassembly of DNA origami were performed reversibly, and dynamic assembly and disassembly were observed on a lipid bilayer surface using HS-AFM. Using a lipid bilayer for DNA origami assembly, it is possible to perform a hierarchical assembly of multiple DNA origami nanostructures, such as the integration of functional components into a frame architecture.

## 1. Introduction

A biological membrane surface, such as a cellular surface, is used for assembling molecules and proteins to function, such as molecular transport, signal transduction, energy conversion, and intercellular communication. To understand such membrane-associated biological events, synthetic biology approaches that design and reconstitute natural biological systems can provide a new insight for elucidating these mechanisms. The lipid bilayer surface has been used for the study of protein assemblies such as membrane-associated proteins and membrane proteins, and their dynamic assembly has been directly observed at nanoscale resolution using atomic force microscopy (AFM) [1,2].

One of the good examples of the molecular assembly system is achieved using DNA molecules that can be directly monitored by AFM. DNA origami that folds DNA into the target 2D and 3D nanostructures is now a standard technology for creating DNA nanostructures [3,4]. The assembling of DNA origami on a lipid bilayer surface is one of the suitable attempts for the surface-assisted biomolecule assemblies, and the research could establish a strategy to control artificially assembled nanostructures using membrane dynamics [5,6].

In addition, in the past two decades, a direct observation of biomolecular movement during reactions and assembly process has been achieved using high-speed AFM (HS-AFM) [2,7,8,9]. The HS-AFM realizes imaging at a subsecond timescale (5–40 frames/s), which enables the real-time imaging of dynamic movements of molecules and their assemblies at a nanoscale resolution [1,2]. This should be applied for the direct visualization of the assembly process of DNA origami and dynamic formation of nanostructures. 

**Figure 1:**
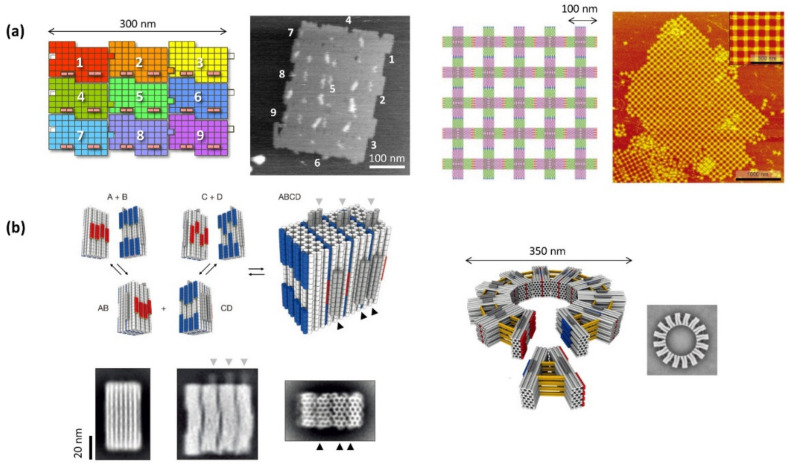
Programmed DNA origami assembly systems. (**a**) Two-dimensional (2D) assembly systems. Nine different DNA origami assembly system using shape- and sequence-complementarity and AFM image of the 3 × 3 assembly (left). Two-dimensional lattice formation by assembling cross-shaped DNA origami using blunt end stacking interaction. (**b**) Three-dimensional (3D) assembly systems. Paired blocks can be connected and assembled in a programmed fashion via shape-complementary and blunt end stacking interaction.

DNA origami is created by folding a long single-stranded DNA (e.g., M13mp18 ssDNA, 7249 bases), referred to as an s scaffold strand, with multiple short complementary DNA strands (staple strands) according to designed shapes (Figure 1a) [3]. The set of staple strands required to build a DNA origami structure contains different sequences to form a double helix at a defined position. Therefore, all of the positions of the formed DNA origami can be addressed using the sequence information of the staple strands. This addressing property is a significant feature of DNA origami, and various molecules can be placed at desired positions in the DNA origami nanostructure by chemically modifying or extending the ends of the staple strands. Therefore, DNA origami is an excellent molecular system that can not only design and construct the desired shapes but also place and arrange the target molecules in the DNA origami nanostructure.

A disadvantage of DNA origami technology is that the size of the DNA nanostructure is limited by the length of the scaffold DNA strand. However, if the designed DNA origami is used as a building block for higher-order nanostructures, a large assembly of more than a micrometer in size can be constructed. The programmed assembly of DNA origami nanostructures into 1D and 2D assemblies was performed using specific hybridization, shape fitting, and π-stacking interactions at the edges (Figure 1b) [10,11]. In addition, the programmed assembly of the DNA origami units into 3D nanostructures was performed by designing the nanostructures and interactions (Figure 1b) [12,13].

## 2. Self-Assembly of DNA Origami on The Lipid Bilayer Surface

Planar DNA origami structures are flexible because of the aligned orientation of the bundled double helices. Therefore, it is complicated to assemble multiple DNA origami structures into a uniform large-scale structure in the solution, and, in many cases, indefinite aggregates are formed. To solve this problem, the surface growth method is employed, in which, molecules are adsorbed and concentrated on the surface of a solid substrate and are two-dimensionally (2D) self-assembled at the solid–liquid interface. Using adsorption on the surface of a substrate, such as mica, the flexibility of the structure is suppressed and the effective concentration is increased; thus, large-scale self-assembly along the surface can be promoted.

For the growth method on a substrate, it is important to set the exact adsorption conditions that allow for the 2D diffusion of molecules on the substrate surface. Most DNA origami is prepared in a neutral buffer solution containing Mg^2+^ at 10–20 mm. However, in such a solution, which is the standard for AFM observation, DNA origami strongly adsorbs onto the surface of the mica substrate, and does not show 2D diffusion. Therefore, for 2D self-assembly using a mica substrate as a support, it is necessary to moderately weaken the adsorption by adding NaCl (approximately a few hundred mM) to the solution [14,15].

However, an approach for changing the support while maintaining the solution conditions is also conceivable. Therefore, a lipid bilayer membrane could be used, which is a biocompatible interface [5]. The mica-supported lipid bilayer has the advantages of providing a flat surface and its fluidity and surface charge being able to be controlled by adjusting the composition of the lipid molecules. Another advantage is that the DNA origami surface (upper side or back side) that makes contact with the lipid bilayer surface can be defined by modifying a specific DNA origami surface with hydrophobic molecules to interact with the lipid. Cholesterol is preferentially used to promote the interaction of DNA nanostructures with the lipid membrane. The construction of an artificial channel has been achieved using hollow DNA origami with multiple cholesterol molecules to anchor onto the membrane surface [16]. The attachment and observation of tubular DNA origami has been carried out using a cholesterol-mediate interaction with the lipid bilayer [17]. In addition, Howorka and coworkers designed and constructed artificial channels using hydrophobic molecules incorporated to the outside center of six helix-bundled DNA tubes to be inserted and penetrate the lipid bilayer of liposomes [18,19,20,21].

**Figure 2:**
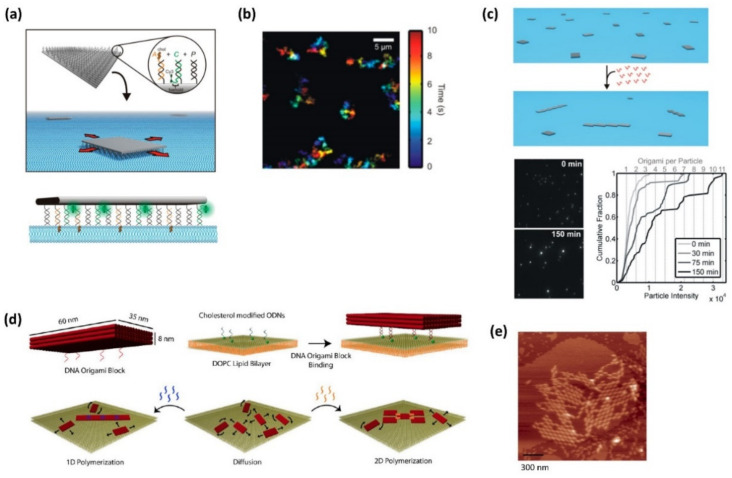
Programmed assembly of DNA origami on a supported lipid bilayer. (**a**) Design of DNA-cholesterol barges. A rectangular DNA origami coated with cholesterol-conjugated anchor strands and Cy3-labeled strands. Cholesterol-conjugated anchors can interact with a lipid bilayer. (**b**) Time-lapse image of DNA barges in a DOPC/DOPE-mPEG lipid bilayer. (**c**) Oligomerization of DNA origami tiles induced by the addition of ssDNA linker strands. TIRF images before and after (150 min) addition of the linker strands. Intensity-weighted cumulative histogram of particle intensities 0–150 min after adding the linker strands. (**d**) Programmed assembly of DNA origami into 1D and 2D assembles by the addition of different connector strands during the DNA origami diffusing on the membrane. (**e**) AFM image of 2D DNA origami assembly on a DOPC lipid bilayer.

Johnson-Buck and coworkers reported a control of the attachment and assembly of cholesterol-conjugated DNA origami (barge) on a supported lipid bilayer for engineering molecular behaviors with membranes rationally (Figure 2a) [22]. DNA barges stably bound onto a lipid bilayer showed 2D Brownian motion, with diffusion coefficients similar to lipid-linked membrane proteins, which was observed using a total internal reflection fluorescence (TIRF) microscopy. The experimental factors, such as cholesterol labels, composition of the membrane, ionic conditions, and surface modification of DNA origami, influence the association to the lipid bilayer and the lateral diffusion. Therefore, the results show that the attachment and detachment from the membrane and oligomerization of DNA barges can be precisely controlled in a programmable manner.

Kocabey and coworkers reported on the membrane-assisted assembly of DNA origami nanostructures that mimic the assembly of membrane-associated proteins [23]. In the study, rectangular three-layered DNA origami blocks (60 nm × 35 nm × 8 nm in size) were attached on a dioreoil phosphatidylcholine (DOPC) lipid bilayer surface using hybridization with anchoring cholesterol-modified DNA strands. DNA origami blocks diffusible on the membrane surface were then assembled into different superstructures, such as end-to-end connection for 1D assembly and corner-to-corner for 2D assembly, by adding different sets of connector strands (Figure 2). Their assembly and morphology changes were observed by AFM. In addition, hexagonal lattice patterns were created by the assembly of triskelion DNA origami assembled using three truncated Y-shaped structures. They also assembled the DNA origami blocks on a small unilamellar vesicle (SUV), and the deformation of the SUVs was observed by the formation of 2D nanostructures and the interaction on the surface. These methods can be used for engineering membrane-embedded molecular systems.

## 3. Large-Sized Self-Assembly of DNA Origami on The Lipid Bilayer Surface

**Figure 3:**
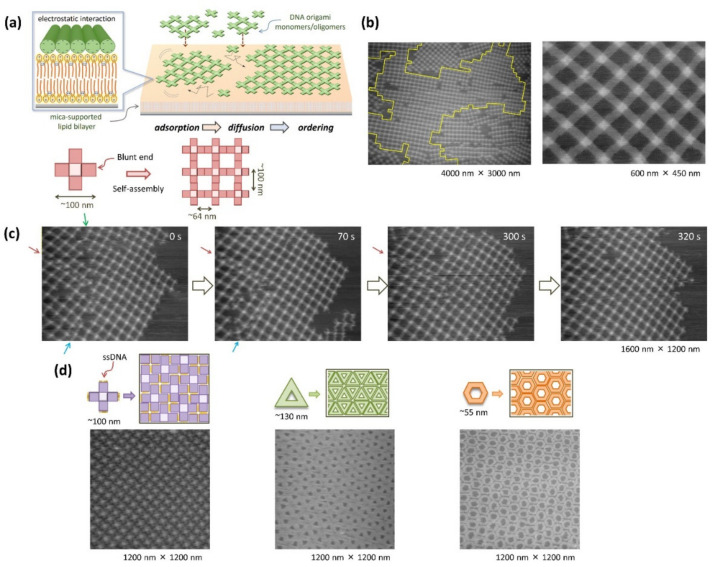
Assembly of DNA origami on a lipid bilayer surface. (**a**) Cross-shaped origami adsorbed and concentrated on the lipid bilayer surface was attached two-dimensionally (2D) by the stacking interaction between their blunt ends to form 2D lattice structures. (**b**) AFM images of the lattice assembled on the lipid bilayer. (**c**) Dynamic process of self-assembly visualized by high-speed AFM (HS-AFM). (**d**) Two-dimensional close-packed assembles formed with cross-shaped (left), triangle-shaped (middle), and hexagon-shaped (right) DNA origami.

Suzuki and coworkers used a cross-shaped DNA origami structure as the component of assembly (Figure 3a) [24]. In this study, a lipid bilayer membrane of DOPC was formed on a mica substrate using a vesicle fusion method, and DNA origami was electrostatically adsorbed onto the membrane surface through cationic Mg^2+^ in solution [25]. The cross-shaped DNA origami concentrated on the lipid bilayer surface associated with each other through π-stacking interactions at the edges of the cross-shaped structure and grew into a micrometer-sized lattice structure (Figure 3b). When this process was observed using HS-AFM [8], large 2D lattice structures were formed on the surface, in which, monomers and multimers consisting of several to several tens of micrometers repeatedly associated and dissociated (Figure 3c). A dynamic rearrangement of DNA origami occurred near the boundary of adjacent lattices, resulting in the formation of a larger lattice structure with uniform orientation. It was also observed that the defects in the grid were repaired by supplemented DNA origami.

In addition, when the same cross-shaped origami structure interfered with the stacking interaction between the edges, the excluded volume effect resulted in a close-packed self-assembly (Figure 3d). Similar aggregate structures could be obtained not only in the cross-shaped DNA origami structure but also in the triangle-shaped and hexagon-shaped origami structures (Figure 3d). Thus, by changing or diversifying the shape of DNA origami and the interaction between DNA origami, it is possible to construct a wide variety of micrometer-sized 2D aggregates on the lipid membrane.

## 4. Continuous Self-Assembly of DNA Origami into The Various Lattices

**Figure 4:**
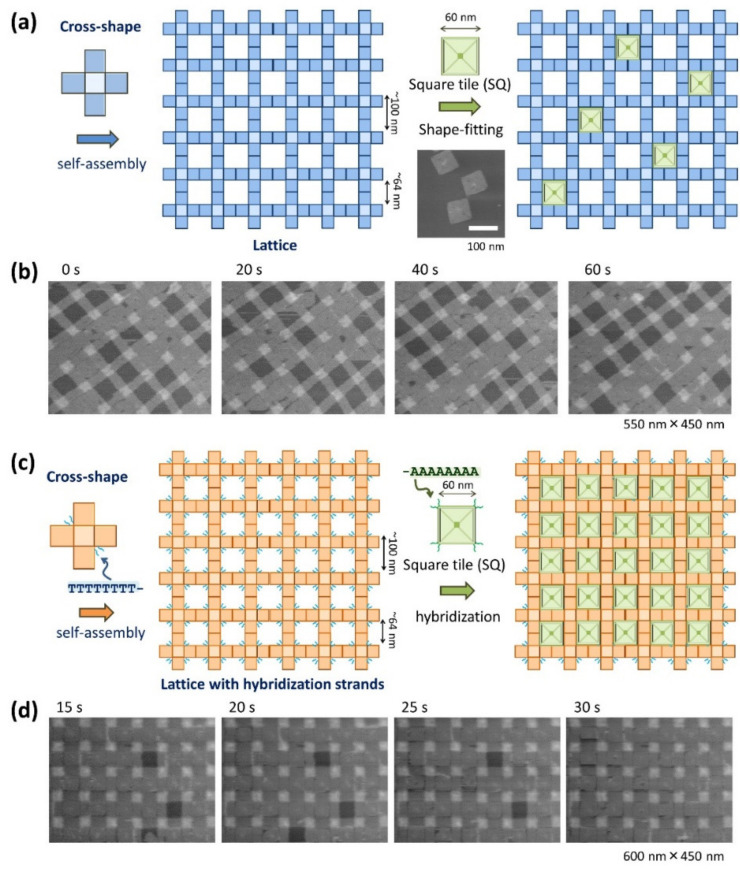
Incorporation of square DNA origami (SQ) tiles into the cavities of a lattice on a lipid bilayer surface. (**a**) After the formation of a lattice by assembling cross-shaped origami, SQ tiles were introduced to fit into the cavities. (**b**) HS-AFM images taken after the SQ tiles are further adsorbed on the lipid bilayer surface on the formed 2D lattice. (**c**) Following the formation of a lattice by assembling cross-shaped origami with T_8_-strands, SQ tiles with A_8_-strands were introduced to hybridize into the cavities. (**d**) HS-AFM images after the SQ tiles are further adsorbed on the lipid bilayer surface on the formed 2D lattice. Scanning rate 0.2 frame/s.

Suzuki and coworkers further examined the combination of a 2D lattice constructed on a lipid bilayer surface with other DNA origami components to build new complex nanostructures (Figure 4) [26]. A square DNA origami tile (SQ tile; 60 nm edge) with a shape complementary to the square cavity (64 nm) of the 2D lattice was prepared. They conducted an experiment in which the SQ tiles were added and adsorbed on the lipid bilayer surface where the lattice formed. The incorporation of the SQ tiles into the lattice cavities was observed, confirming the shape-fitting of DNA origami structures. At low Mg^2+^ concentrations (10 mM), the SQ tiles repeatedly attached and detached to the cavities (Figure 4b). Furthermore, complementary DNA strands were incorporated into both the cross-shaped origami and SQ tile to tightly associate the tiles into the lattice via hybridization (Figure 4c). DNA strands (T_8_-strands) were introduced to the cross-shaped DNA origami to modify inside the lattice cavities, and complementary DNA strands (A_8_-strands) were incorporated into the four corners of the SQ tile. After the addition of the SQ tiles to the lattice, the SQ tiles can hybridize on the lipid bilayer surface, and the SQ tiles stably attached inside the lattice cavity (Figure 4d). During HS-AFM scanning, a dissociation and association of the SQ tile to the lattice were observed, but the frequency was very low.

**Figure 5:**
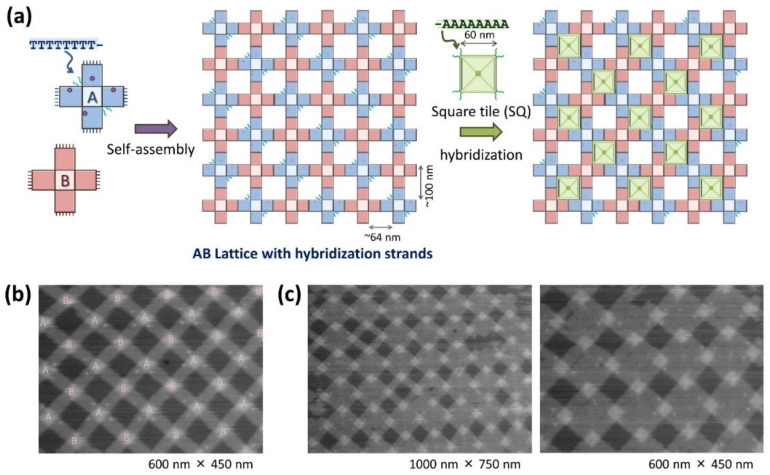
Formation of a checkered pattern using AB-lattice and SQ tile. (**a**) Assembly of AB-lattice and incorporation of SQ tile into the specific cavities. T_8_-strands introduced in Cross-A hybridize with A_8_-strands introduced to SQ tile. (**b**) AFM image of AB-lattice. In order to distinguish Cross-A and Cross-B, Cross-A was modified with biotins to be labelled by streptavidin. (**c**) Incorporation of SQ tiles into the formed AB-lattice. AFM images of SQ-attached AB-lattice after incorporation of SQ tiles.

Using this lattice–tile system, the lattice with SQ tiles was diversified into a checkered pattern (Figure 5a). First, two types of cross-shaped DNA origami structures (Cross-A and Cross-B) were prepared and assembled to construct an AB lattice. T_8_-strands were introduced into the Cross-A origami, and then the AB-lattice was formed with unmodified Cross-B. Following AB-lattice formation, an alternative assembly of Cross-A and Cross-B was observed from the labelled Cross-A in the lattice in the AFM image (Figure 5b). Subsequently, SQ tiles with complementary A_8_-strands were added to the AB lattice cavities. The SQ tiles were captured into specific cavities through hybridization (Figure 5c). When the SQ tiles entered a cavity without complementary strands, they rapidly dissociated from the cavity. Using this mechanism, the SQ tiles were incorporated into the predesigned grid to associate them into a checkered pattern.

## 5. Assembly on a Phase-Separated Lipid Bilayer Surface

**Figure 6:**
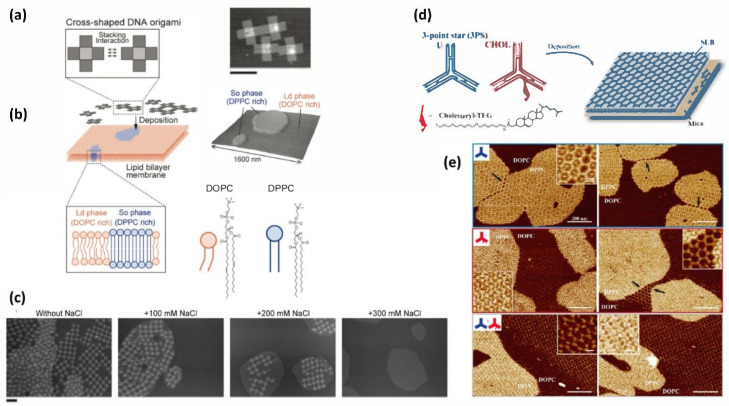
Formation of 2D assemblies on a phase-separated membrane. (**a**) Cross-shaped DNA origami was deposited onto a lipid membrane. (**b**) DOPC forms liquid-disordered (Ld) phase and DPPC forms solid-ordered (So) phases. AFM image of cross-shaped origami (scale bar: 100 nm) and phase-separated membrane on the mica surface. (**c**) AFM images of cross-shaped DNA origami adsorbed on DOPC/DPPC phase-separated membrane surface at different NaCl concentrations (0–300 mM). (**d**) Assembly of 3-point star tile (U) and cholesterol-modified tile (CHOL) on a DOPC and DPPC surface. (**e**) AFM images of assemblies of U tile (top), CHOL tile (middle), and U/CHOL mixture (bottom) on the DOPC/DPPC phase-separated membrane surface.

In cellular membranes, multiple lipids form specific domains, called a raft, to regulate biological processes through the organization of biomolecules into these domains [27,28,29]. A unique phenomenon that occurs in lipid bilayers is the phase separation that leads to the formation of specific domains. Sato and coworkers controlled the assembly of the DNA origami nanostructures using fluidic and gel-phase lipids [30]. By self-association of cognate lipids, the mixture of unsaturated DOPC and saturated DPPC (1,2-dipalmitoylsn-glycero-3-phosphatidylcholine) forms a phase-separate membrane with a fluidic liquid-disordered (Lo) phase and solid-ordered (So) phase, respectively (Figure 6a,b). The assembly of DNA origami on the membrane was observed using AFM. When a blunt-ended DNA cross-shaped origami was added onto the DOPC/DPPC phase-separated surface, DNA origami preferentially bound to the So phase. As a result of the higher charge density and lower fluidity of the So phase, the cross-shaped origami formed packed assemblies, and the surface mobility of the origami was restricted compared with that of the Ld phase. At higher concentrations of DNA origami, 2D lattices were formed on the Ld phase through surface-mediated dynamic assembly. The movement of origami adsorbed on the So phase was suppressed and the origami packed as aggregates (Figure 6c). Through the addition of NaCl, which can detach the origami from the membrane surface, the origami lattice formed on the Ld phase detached from the surface and the packed aggregates on the So phase reorganized into the lattices. The results indicate that the formation of 2D assemblies depends on the phase of the lipids and that a higher ion concentration promotes the reorganization of the 2D assemblies.

Avakyan and coworkers carried out the controlled assembly of a small three-point star DNA tile into a 2D hexagonal array on a lipid bilayer surface (Figure 6d) [31]. Using cholesterol-anchoring and stacking interactions, DNA tiles selectively formed 2D arrays on the mica, fluidic DOPC, and gel-state DPPC surface. Using an unmodified DNA tile (U) and DOPC surface, a hexagonal array formed only on the mica, whereas the U tile was found to form a hexagonal array on the gel-state DPPC surface because of the densely packed DPPC lipid. In the case of using cholesterol-modified tile (CHOL), a CHOL tile formed a hexagonal array both on the DOPC and DPPC surface because of the interaction of the lipids and cholesterol molecule. Using the phase-separated state of the mixture of DOPC and DPPC, CHOL formed a hexagonal lattice on DOPC, and the U and CHOL mixture formed a densely packed array on DPPC. The array patterns can be controlled using cholesterol and assemble on the phase-separated lipid bilayer surface.

## 6. Photo-Controlled Assembly and Disassembly of DNA Origami

**Figure 7:**
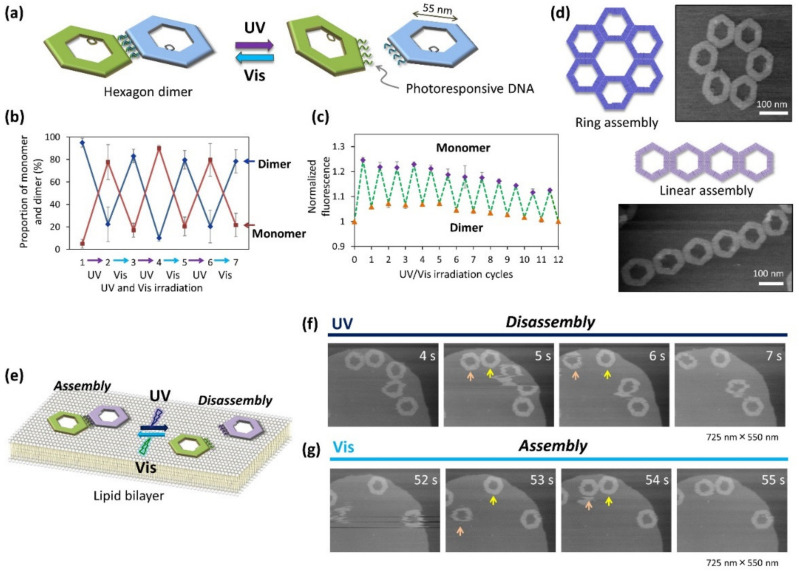
Direct observation of the assembly and disassembly of photoresponsive hexagon DNA origami. (**a**) Assembly and disassembly of hexagon origami carrying four photo-switching DNA strands by UV/Vis irradiation. (**b**) Dimer formation and dissociation of hexagon origami by alternating UV/Vis irradiation, which was quantified by gel electrophoresis. (**c**) Dynamic dimer formation and dissociation of hexagons in solution by alternating UV/Vis irradiation, which was characterized by fluorescence quenching. (**d**) Formation of ring-shaped and linear hexagon assemblies and their AFM images. (**e**) Observation of dynamic assembly and disassembly on a lipid bilayer surface by UV/Vis irradiation. (**f**,**g**) Direct HS-AFM observation of disassembly of the dimer on the lipid bilayer surface during UV irradiation (**f**) and assembly of the monomers during Vis irradiation (**g**). Arrows indicate the dissociated and associated hexagon origami. Scanning rate 1 frame/s.

Suzuki and coworkers performed a controlled DNA origami assembly using photoreactions. The advantage of using a photoreaction is that the interaction can be initiated and controlled by irradiation at a specific wavelength of light. Photoswitching DNA strands containing azobenzene molecules have been used to observe single-molecule dissociation and hybridization in DNA origami scaffolds using HS-AFM [32]. In addition, photoswitching DNA strands have been used for the assembly and disassembly of DNA origami nanostructures [33]. Hexagonal DNA origami bearing four photoswitching DNA strands was used. The hexagon dimer in the initial state (trans-azobenzene) was dissociated into monomers (cis-azobenzene) using UV irradiation, and then reassembled to form a dimer by visible light (Vis) irradiation (Figure 7a). The assembly and disassembly were reversibly controlled by UV/Vis irradiation in solution. Dimer formation and dissociation in the solution were examined using gel electrophoresis (Figure 7b), and dynamic formation and dissociation in the solution were monitored using fluorescence quenching (Figure 7c). The repeated assembly and disassembly of hexagons was observed in high yields by alternating UV/Vis irradiation. Various assemblies, such as linear and ring-shaped nanostructures, were formed by changing the positions of the photoswitching DNA strands in the hexagonal origami (Figure 7d).

Using this photoresponsive DNA origami system, the dynamic assembly and disassembly of hexagonal DNA origami on a lipid bilayer surface was directly observed using HS-AFM (Figure 7e) [34]. The fluidic properties of a lipid bilayer can be altered by adjusting the lipid composition according to the target molecules and structures [5]. Cholesterol moieties were introduced into the hexagon origami to control the interaction with the lipid. The cholesterol-modified photoresponsive hexagon origami dimers were loaded onto the DOPC/sphingomyelin phase-separated lipid bilayer and their diffusion and interactions were observed using HS-AFM. The diffusion of hexagonal DNA origami is needed to be deaccelerated for the AFM observation. The cholesterol-modified hexagonal origami attached on the gel-phase sphingomyelin surface can be visualized with moderate diffusion. When these photoresponsive hexagonal dimers in the initial state were exposed to UV light during AFM scanning, the dimer immediately dissociated into monomers (Figure 7f). Subsequently, by irradiating Vis light to the monomers diffusing on the surface, two monomers attached at the correct edges to form a dimer after diffusion and collision (Figure 7g). The dimer formed was dissociated again by the second round of UV irradiation. The reversible assembly and disassembly of the hexagon origami was directly visualized on the lipid bilayer surface. These photo-controlled manipulations of the interactions of DNA nanostructures can be expanded to the construction of various switching nanodevices.

## 7. Conclusions and Perspectives

One of the solutions to construct micrometer-sized DNA assemblies is to utilize a lipid bilayer. Using a mica-supported lipid bilayer, DNA origami nanostructures can be further formed into micrometer-sized large assemblies. Large lattices were formed from DNA origami on a membrane surface, and their formation was controlled by the addition of connection DNA strands. In addition, the dynamic behavior of the lattice formation was observed using HS-AFM. The attachment and detachment of the cross-shaped DNA origami to the formed lattice were visualized, and the rearrangement of the lattice can be observed in real time. Furthermore, various types of formed lattices were used to assemble DNA origami tiles into their cavities on the membrane surface. Various patterns of lattice–tile complexes were designed and programmed by changing the interaction between the lattice cavity and the DNA tiles. Using unique properties of phase-separated lipid bilayers, the formation of assemblies of DNA origami can be controlled specifically. For the further switching of the assembly and disassembly of DNA origami, a photo-controlled assembly system was created using photoswitching DNA strands. By alternatively irradiating UV and Vis light, the detachment of the dimer and attachment of monomers were controlled and directly observed on a lipid bilayer using HS-AFM.

Using these strategies employing a lipid bilayer for DNA origami assembly, it is possible to perform the hierarchical assembly of multiple DNA origami nanostructures, such as the incorporation of functional components (guests) into a frame architecture (host). For example, designed nanoscale compartments partitioned by a DNA lattice can be constructed to create an array of DNA tiles with membrane proteins, such as a channel and receptor [35]. It is possible to periodically place multiple types of DNA origami tiles into the lattices, as well as to arrange the spacing and positions of these functional tiles in the lattices. This can be applied as a platform for constructing cascade reactions with multiple enzymes that mimic molecular systems in cells.
